# Prognostic Factors and Optimal Response Interval for Stereotactic Body Radiotherapy in Patients With Lung Oligometastases or Oligoprogression From Colorectal Cancer

**DOI:** 10.3389/fonc.2019.01080

**Published:** 2019-10-15

**Authors:** Shuai Li, Dezuo Dong, Jianhao Geng, Xianggao Zhu, Chen Shi, Yangzi Zhang, Hongzhi Wang, Shun Zhou, Hao Wu, Yong Cai, Yongheng Li, Weihu Wang

**Affiliations:** Key Laboratory of Carcinogenesis and Translational Research (Ministry of Education/Beijing), Department of Radiation Oncology, Peking University Cancer Hospital and Institute, Beijing, China

**Keywords:** colorectal cancer, oligometastases, oligoprogression, stereotactic body radiotherapy, biologically effective dose, gross tumor volume, evaluation interval

## Abstract

**Purpose:** To analyze the prognostic factors and optimal response interval for stereotactic body radiotherapy (SBRT) in patients with lung oligometastases (OM) or oligoprogression (OP) from colorectal cancer (CRC).

**Method:** Patients with lung OM or OP from CRC treated by SBRT at our hospital were included in this retrospective review. The local control (LC), response to SBRT in different evaluation interval and regional metastases (RM) was analyzed. The risk factor for LC and RM was calculated using the Kaplan-Meier method and compared using the Log-rank test. Multivariate analysis with a Cox proportional hazards model was used to test independent significance.

**Results:** A total of 53 patients with 105 lung metastases lesions treated from 2012 to 2018 were involved in this retrospective study. The median biologically effective dose (BED) for these patients was 100 Gy (range: 75–131.2 Gy). Complete response (CR) increased from 27 (25.7%) to 46 (43.8%) lesions at 1.8 and 5.3 months following SBRT, and at the last follow-up, 52 (49.5%) lesions achieved CR. The median follow-up duration for all patients was 14 months (range: 5–63 months), and 1-year LC was 90.4%. During the follow-up, 10 lesions suffered local relapse after SBRT (9 of them occurred within 8 months after SBRT). The univariate analysis shows BED ≥ 100 Gy (*P* = 0.003) and gross tumor volume (GTV) < 1.6 cm^3^ (*P* = 0.011) were better predictors for 1-year LC. The patients with lung oligoprogression had higher 1-year RM when compared with patients with lung oligometastases (hazard ratio 2.78; 95% confidence interval [CI] 1.04–7.48, *P* = 0.042). Until the last follow up, 4 (7.5%) patients suffered grade 2 radiation pneumonitis, and no grade 3–4 toxicity was observed.

**Conclusions:** SBRT provides favorable LC in CRC patients with lung OM or OP, and the GTV and BED can affect the LC. Radiology examinations nearly 5–6 months following SBRT appear to represent the final local effect of SBRT, and the patients with oligoprogression has higher RM.

## Introduction

Metastases are observed in 20% of all colorectal cancer (CRC) patients at diagnosis, and up to 50% patients will develop metachronous metastases during the period of whole treatment and follow-up (1). The lung is one of the most common site of metastases for CRC (2). Until now, chemotherapy remains the standard treatment for lung metastases (LM) from CRC. Although chemotherapy can prolong the survival time of patients, long-term survivors are extremely rare. Many studies have shown that additional local treatments of LM from CRC after radical surgery of primary disease may confer a survival benefit (3, 4). Routinely surgical resection was considered to be the most acceptable local treatment of LM from CRC (5, 6). However, due to heterogeneity of disease or patient, high percentage of patients are not suitable for radical resection, therefore the alternative local approaches are urgently needed.

As a high-precision radiation technology, stereotactic body radiotherapy (SBRT) uses higher doses and less fraction to treat isolated sites of cancer. In none-small-cell lung cancer, SBRT has gained favorable local control (LC) and overall survival (OS) when compared with surgery. Meanwhile it is well-tolerated and can be safely administered with low morbidity (7). Since 1995, oligometastases has been presented to describe an intermediate state of cancer spread that lies between localized disease and widespread metastases. For patients with oligometastases, the disease-free survival (DFS) and OS can both be improved if LC is achieved (8–10). Another newly indication for SBRT is the status of “oligoprogression.” This scenario means the cancer progression occurs in a limited number of tumors, while the other metastases are stable or responding to systemic therapy strategy. For patients with oligoprogression, SBRT can prolong the progression-free survival (PFS) and delay changes of chemotherapy schedules (11).

Some studies were performed to test the effect of SBRT in the treatment of patients with lung OM or OP from CRC, and revealed that 1-year LC rate was 85–95%. Most of the studies reported that dose escalation could affect the LC rate after SBRT (12–16). Meanwhile, advances in imaging diagnosis now allow the detection of metastases <1 cm, leading to an increased number of patients with tiny LM from CRC at the initial diagnosis or regular follow-up after radical surgery of primary CRC. However, whether high dose is necessary for these tiny metastases remains unclear. Additionally, in clinical practice, we frequently confront the questions raised by the patients that when and how to perform the radiation evaluation, and how to deal with residual LM or another newly emerged LM (defined as regional metastases in our study) after first course of SBRT for OM or OP. Thus, we performed this retrospective study to analyze the risk factors that affect the LC or regional metastases, and optimal response interval for CRC patients with lung OM or OP treated by SBRT.

## Materials and Methods

### Patients

We retrospectively reviewed medical records of patients treated at the Peking University Cancer Hospital and Institute, Beijing, China. Patients were eligible for inclusion if (1) they had histopathologically confirmed primary CRC and had receive radical surgery for the primary tumor; (2) they was pathologically or radiologically confirmed lung metastases during treatment; (3) they had Eastern Cooperative Oncology Group performance status (ECOG-PS) of 0–2; (4) the SBRT treatment has potential therapeutic implications decided by multidisciplinary treatment (MDT) group; (5) they had completed the whole SBRT regimen plan and had sufficient details of other treatment and follow-up. Patients with LC after a previous course of SBRT were allowed to undergo repeat SBRT for new lesions. All patients were stratified into two groups: those with total metastatic lesions <5 and with a maximum tumor diameter of 50 mm were defined as oligometastases group, and other patients with more than five metastatic lesions but with <5 progressed lesions(or newly occurred) limited in lung during systemic therapy were categorized as oligoprogression group.

Before SBRT, each patient signed informed consent after receiving an explanation for possible benefits and complications, and approved the involvement of the treatment and follow-up. The study was conducted in accordance with the Declaration of Helsinki, and the protocol was approved by the Ethics Committee of Peking University Cancer Hospital and Institute (No. 2019YJZ40).

### Radiotherapy Technique

All patients were immobilized with a thermoplastic mask or vacuum cushion device. The four-dimensional computed tomography (4D-CT) simulation technique (slice thickness of 3 mm) was implemented for all patients. For patients who had received 4D-CT, the gross tumor volume (GTV) was identified in the lung parenchyma window. The internal gross tumor target volume (IGTV) was defined as the phase of maximum intensity projection reconstructed by each phase, and the final planning treatment volume (PTV) was defined as the IGTV plus an isotropic margin of 5 mm according to the experience of our department. Organs at risk (OAR) was delineated mainly according to Kong's study with minor adjustments (17).

The radiotherapy plan was calculated on the CT average image set, and patients were treated with the Intensity Modulated Radiation Therapy (IMRT) or volumetric modulated arc therapy (VMAT) technique. The 95% isodose line was required to cover the PTV, and any dose >105% of the prescribed dose should not occur outside the PTV. The dose-fractionation schemes were prescribed by the treating physicians depending on the tumor volume, location, and dose constrains of normal tissues. Dose volume histograms (DVH) was calculated for any irradiated vital organs including the lung, heart, spinal cord, chest wall, liver, stomach and esophagus. Dose constraints for OAR were defined based on the recommendations of RTOG-0236 with minor adjustments when needed (18). The SBRT was delivered daily for 5 days within 1 week. The online CBCT-based volumetric image-guided radiation therapy using soft tissue registration for target was applied before all SBRT fractions deliveries.

### Outcome Evaluation

Patients were evaluated by the treating physician weekly during the treatment. Additional follow-up visit and radiological evaluation were planned at nearly 1 month following the completion of SBRT and every 3 months after that for 2 years. The radiological evaluation including CT, positron emission tomography–computed tomography (PET-CT) scans, and tissue biopsy if necessary. The treated lesions were assessed by one diagnostic radiologist and one radiation oncologist on each scan. Tumor response was defined according to the modified version of the RECIST (Response Evaluation Criteria in Solid Tumors) criteria (19). Synchronous oligometastases was defined as the interval between presence of lung metastasis and initial diagnosis not exceeding 6 months. Local failure differs from a complete response (CR) in that tumor completely shrinkage is not required, but there must never be any re-growth within the PTV. Regional failure is defined as a newly developed lung metastases within the thorax and outside the field of PTV.

The primary endpoint was LC, and the secondary endpoints included OS and regional metastases (RM). LC was measured from the date of the first course of SBRT to the date of local failure, OS was measured from the date of the first course of SBRT to the date of death for any cause or date of final follow-up, and RM was measured from the date of the first course of SBRT to the date of regional failure.

Acute toxicity was graded according to the Common Terminology Criteria of Adverse Events Version 4.0 (CTCAE V4.0), and late toxicities (≥6 months after radiation therapy) were evaluated using the late toxicity scoring system of Radiation Therapy Oncology Group (RTOG).

### Statistical Analyses

Biologically effective dose (BED) was calculated using the linear quadratic formula: BED_10_ = nd × (1 + d/[α/β]), where “n” represent the number of fractions, “d” represent the dose/fraction, and “α/β” is equal to 10Gy (20). Data were collected and analyzed using Statistical Package for the Social Sciences (IBM Corp., SPSS Statistics for Windows, v. 22.0. Armonk, NY; USA). The analysis of LC was conducted at lesion's level. The distribution of GTV and BED for each lesion was analyzed using R software (version 3.2.3; http://www.r-project.org/). The χ^2^ test were used to compare the differences between the 2 groups. The LC, RM and OS were evaluated using the Kaplan-Meier method, and the Log-rank test was used to compare survival outcomes of different groups. Multivariate analysis with a Cox proportional hazards model was used to test independent significance. Hazard ratios (HRs) and 95% confidence intervals (CIs) were generated. *P* < 0.05 was considered as statistical significance.

## Results

### Patient and Tumor Characteristics

From August 2012 to August 2018, a total of 53 eligible patients and 105 lung metastases lesions with median tumor diameter of 11 mm (range: 5–40 mm) and median tumor volume of 1.6 cm^3^ (range: 0.3–51.8 cm^3^) were selected and analyzed (Table 1). Of those 53 patients, 13 patients were classified as oligoprogression and 40 patients were classified as oligometastases. In total, 43 patients (81.1%) had received chemotherapy before SBRT (13/13 in oligoprogression group and 30/40 in oligometastases group). Of those 43 patients received chemotherapy, four (9.3%) had a partial response, 21 (48.8%) had stable disease and 18 (41.9%) had progression of disease according to RECIST criteria. Also, 15 patients had received target therapy, 9 patients had received radical resection for lung metastases and suffer newly LM before SBRT.

**Table 1:** Clinical characteristics for 53 patients/105 lesions.

**Variable**	**Value (%)**
Age, median (range)	61 years (40–84 years)
**Sex**	
Male	43 (81.1%)
Female	10 (18.9%)
**Primary disease**	
Colon	8 (15.1%)
Rectal	45 (84.9%)
**ECOG**	
0	21 (39.6%)
1	22 (41.5%)
2	10 (18.9%)
**Response for chemotherapy (43 pts)**	
PR	4 (9.3%)
SD	21 (48.8%)
PD	18 (41.9%)
Time from primary diagnosis to lung metastases, median (range)	19.6 m (0–40 m)
**Chemotherapy before SBRT**	
Yes	43 (81.1%)
No	10 (18.9%)
Cycles (range)	8 (1-21)
**KRAS status**	
Wild type	15 (28.3%)
Mutated	16 (30.2%)
Unknown	22 (41.5%)
**Synchronous metastases**	
Yes	5 (9.4%)
No	48 (90.6%)
**Microsatellite instability**	
MSI	2 (3.8%)
MSS	31 (58.5%)
Unknown	20 (37.7%)
**Metastases subgroup**	
Oligometastases	40 (75.5%)
Oligoprogression	13 (24.5%)
**Treated lesion number**	
1	25 (47.1%)
2	14 (26.5%)
3	4 (7.5%)
4	10 (18.9%)
GTV (cm^3^, range)	1.6 (0.3–51.8) cm^3^
Diameter, mm (Range)	11 mm (5–40 mm)
PTV(cm^3^, range)	17.5 (3.7–149.1) cm^3^
Ratio of PTV/GTV (range)	9.3 (2.5–38.3)
**Total course of SBRT**	
1	43 (81.1%)
2	10 (18.9%)
**SBRT dose prescription (105 lesions)**	
75 Gy/10F	1 (0.9%)
63 Gy/9F	4 (3.8%)
60 Gy/10F	17 (16.2%)
60 Gy/8F	20 (19.1%)
50–55 Gy/5F	33 (31.4%)
48–50 Gy/4F	12 (11.4%)
50 Gy/10F	18 (17.1%)
Time from metastases to SBRT, median (range)	7.9 m (0.5–40.8 m)
**Lesions location (105)**	
Upper lobe	46 (43.8%)
Middle lobe	18 (17.1%)
Lower lobe	41 (39.1%)
**Chemotherapy after SBRT**	
Yes	15 (28.3%)
No	38 (71.7%)

After conversion of dose according to BED_10_, the median BED value for all 105 lesions was 100 Gy. For 36 lesions whose BED_10_ < 100 Gy, 22 lesions were near the chest wall and rib, 8 lesions were near the bronchial tree, 3 lesions were located in the left lung of one patient simultaneously, 2 lesions were near the esophagus, and 1 lesions due to the patient had received previous SBRT.

### Interval for Response Evaluation

The median period from SBRT to the initial radiology evaluation was 1.8 months (range: 0.5–8.0 months). For all 105 lesions, 27 lesions (25.7%) achieved CR. Then the second radiology evaluation, 5.3 months (range: 1.9–12.5 months) after SBRT, showed 46 lesions (43.8%) achieved CR (Table 2). For the 76 PR/SD lesions at initial evaluation, 19 lesions turn into CR at second evaluation. Among them, 6 lesions (31.6%) of 5 patients had received chemotherapy after SBRT, and whether chemotherapy didn't affect the conversion rate from PR/SD to CR (*P* = 0.770).

**Table 2:** The distribution of radiology evaluation by RECIST criteria.

**Radiology evaluation interval**	**CR (%)**	**PR (%)**	**SD (%)**	**PD (%)**
**FOR 105 LESIONS**				
Initial evaluation (1.8 months)	27 (25.7%)	46 (43.8%)	30 (28.6%)	2 (1.9%)
Second evaluation (5.3 months)	46 (43.8%)	38 (36.2%)	12 (11.4%)	9 (8.6%)
Last follow-up	51 (48.6%)	28 (26.7%)	16 (15.2%)	10 (9.5%)

*The bold values means P <0.05, which was considered as statistical significance*.

By the end of the last follow-up, a total of 52 lesions (49.5%) achieved CR evaluated by radiology examinations, and 10 lesions (9.5%) suffered progress disease (PD) (Table 2). For 44 lesions with residual lesions (PR/SD), median time from SBRT to the last follow-up was 20 months (5.1–63.0 months), and no in-field progression were found by radiology examinations.

### Local Control at Lesions Level

**Figure 1:**
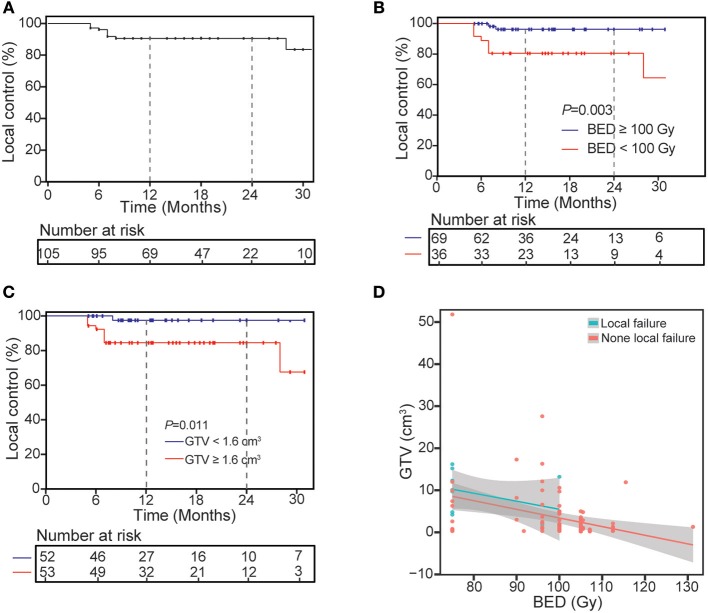
Kaplan-Meier curves of local control for treated lesions **(A)** and univariate analysis of correlation between characteristics with local control **(B,C)** and the distribution of GTV and BED for all lesions divided into two groups **(D)**.

At the time of analysis, median follow-up time for all patients was 14 months (range: 5–63 months), and 1-year LC was 90.4%. At last, 10 lesions suffered local relapse after SBRT, 9 of them occurred within 8 months post-SBRT, and median time to local recurrence was 7 months (range: 5–28 months). Univariate analysis of the correlation between characteristics of 105 lesions and LC revealed BED (96.3% for BED ≥ 100 Gy vs. 80.6% for BED <100 Gy, *P* = 0.003) and GTV (84.5% for GTV ≥ 4.5%^3^ vs. 97.6% for GTV <1.6 cm^3^, *P* = 0.011) can influence the 1-year LC (Table 3 and Figure 1). After multivariable analysis, Higher BED (≥100 Gy) was a better predictor of LC (HR 0.20, 95% CI 0.04–0.98; *P* = 0.047). The GTV had a negative correlation with BED, and in the BED ≥ 100 Gy group, the larger GTV was rare. Meanwhile, for patients who suffered local recurrence, most of them had large GTV and low BED simultaneously, and whether chemotherapy before (*P* = 0.123) or after (*P* = 0.472) SBRT has no significant impact on local control.

**Table 3:** Univariate and multivariable analysis for local control (LC) of 105 lesions.

	**Univariate analysis**		**Multivariate analysis**		
	**1-year LC**	***P***	**HR**	**95% CI**	***P***
**AGE**					
<61	97.5%	0.065			
≥61	84.5%				
**SEX**					
Male	90.2%	0.664			
Female	92.9%				
**SYNCHRONOUS METASTASES**					
Yes	100%	0.208			
No	89.1%				
**CHEMOTHERAPY BEFORE SBRT**					
Yes	87.0%	0.123			
No	100%				
**LOCATION OF LESIONS**					
Upper lobe	90.9%	0.925			
Middle lobe	94.1%				
Lower lobe	88.5%				
**CHEMOTHERAPY AFTER SBRT**					
Yes	92.7%	0.472			
No	89.8%				
**METASTASES SUBGROUP**					
Oligometastases	93.2%	0.312			
Oligoprogression	85.7%				
**BED**					
≥100 Gy	96.3%	**0.003**	0.20	0.04–0.98	**0.047**
<100 Gy	80.6%				
**GTV**					
≥1.6 cm^3^	84.5%	**0.011**	5.80	0.71–47.38	0.101
<1.6 cm^3^	97.6%				

### Regional Metastases and Overall Survival for Patients

**Figure 2:**
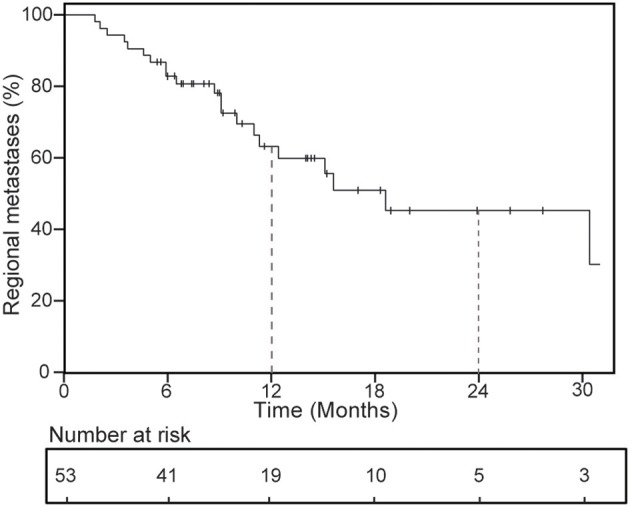
Kaplan-Meier curves of regional metastases for all patients.

For 53 patients included in the analysis, 21 patients suffered regional metastases at the last follow-up, and 10 patients had received second course of SBRT after the occurrence of regional metastases. Rates of regional metastases at 1 year were 36.9% (Figure 2), number of lesion treated (14.9% for 1 lesion vs. 51.3% for 2 or more lesions, *P* = 0.009) and metastases subgroup (25.1% for oligometastases vs. 79.5% for oligoprogression, *P* = 0.001) impacted on 1-year regional metastases (Table 4). After multivariable analysis, oligoprogression was also a worse predictor of RM (HR 2.78, 95% CI 1.04–7.48; *P* = 0.042).

**Table 4:** Univariate and multivariable analysis for regional metastases (RM) of 53 patients.

	**Univariate analysis**		**Multivariate analysis**		
	**1-year RM**	***P***	**HR**	**95% CI**	***P***
**AGE**					
<61	21.7%	0.852			
≥61	46.2%				
**SEX**					
Male	36.8%	0.974			
Female	30.0%				
**CHEMOTHERAPY BEFORE RT**					
Yes	42.0%	0.977			
No	10.0%				
**KRAS STATUS**					
Wild type	38.7%	0.757			
Mutated	51.8%				
Unknown	27.6%				
**CHEMOTHERAPY AFTER RT**					
Yes	53.3%	0.332			
No	34.4%				
**SYNCHRONOUS METASTASES**					
Yes	60.0%	0.117			
No	33.8%				
**BED**					
≥100 Gy (all lesions)	34.7%	0.338			
<100 Gy (at least one lesion)	40.3%				
**NUMBER OF LESION TREATED**					
1	14.9%	**0.009**	2.63	0.79–8.78	0.114
≥2	51.3%				
**METASTASES SUBGROUP**					
Oligometastases	25.1%	**0.001**	2.78	1.04–7.48	**0.042**
Oligoprogression	79.5%				

*The bold values means P <0.05, which was considered as statistical significance*.

**Figure 3:**
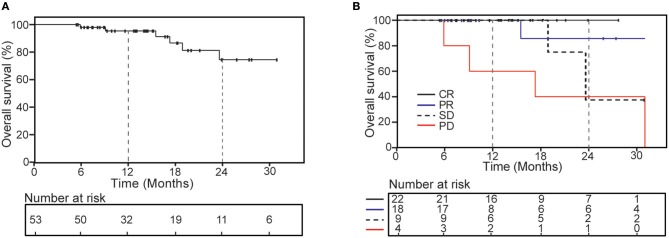
Kaplan-Meier curves of overall survival for all patients **(A)** and according to the second radiology evaluation response **(B)**.

The 1- and 2-years OS was 95.5 and 74.5% (Figure 3), respectively. The second radiology evaluation had significant correlation with 2-years OS (CR vs. PR vs. SD vs. PD: 100 vs. 85.7 vs. 53.3 vs. 25%; *P* = 0.006). Nine patients died, 5 of them experienced complication by multiple lung metastases, and 4 died from metastases of other sites outside of lung (2 to brain, 1 to peritoneal and 1 to liver).

### Toxicity

Grade 3 or higher toxicities were not observed in all these 53 patients, and no one suffered treatment interruption due to SBRT. Four patients suffered grade 2 radiation pneumonitis (1 received 2 courses of SBRT, 2 patients received SBRT for multiple lesions, 1 patient with PTV more than 77.5 cm^3^). Acute hematologic toxicities occurred in 4 patients, those included grade 2 thrombocytopenia (*n* = 1), grade 1 leucopenia (*n* = 2), and grade 1 thrombocytopenia (*n* = 2). The majority of grade 1 toxicity was in the form of self-limiting fatigue (*n* = 12) and esophagitis (*n* = 7). No patient developed severe chest pain or rib fracture until to the last follow-up.

## Discussion

In this study, SBRT can obtain an effective LC and acceptable toxicities for patients with OM or OP from CRC. High doses (BED_10_ ≥ 100 Gy) and small lesions volume (GTV <1.6 cm^3^) appear to be associated with better LC. After SBRT, the second course of radiology evaluation maybe a better predictor for LC and OS, which was the main aim for the lung OM or OP from CRC. We also revealed the biological advantage of oligometastases status had significantly lower RM.

The lung ranks the second most common metastatic site after the liver for CRC. Though chemotherapy is frequently used in clinical practice for patients with LM from CRC, local treatment for selected patients with suitable status may increase the possibility of a cure and long survival. A study containing 544 CRC patients with LM shows that 44 patients receiving local therapy, such as surgery or radiotherapy had significantly longer median PFS (16.1 vs. 4.7 months; *P* < 0.001) and OS (51.8 vs. 23.5 months; *P* < 0.001) than those treated with chemotherapy alone (3). Recently, an analysis based on pooled data of 500 metastatic lesions (lung = 209, liver = 291) from 388 CRC patients revealed that median survival time of all patients was 27.9 months, and the median survival time for patients with and without local failure after SBRT was 25.4 vs. 30.6 months (21). In our study, the 2-years OS for all patients was 74.5%, and the curative effect of SBRT could influence the OS, which indicated that patients with OM or OP from CRC could obtain good prognosis if received suitable SBRT treatment schedule.

For the patients with LM from CRC who received SBRT, it is widely believed that the biologically effective dose may influence the efficacy of radiotherapy. A meta-analysis was conducted to analyse the prognosis of 1,920 patients with pulmonary oligometastases, and better LC was achieved by a higher prescription dose than lower prescription dose (odds ratio = 0.16, *P* < 0.001) (22). Meanwhile, some studied indicated the tumor size may affect the effect of LC (23, 24). For example, Kang et al. evaluated 59 CRC patients with 78 lesions confined to one organ, whose median prescription radiation dose was 42 delivered in 3 fractions (BED_10_ = 100.8 Gy), they observed that smaller GTV was a significantly favorable prognostic factor for LC (23). In our study, 1-year LC was 90.4% for 105 lesions, when compared with the patients in other studies, our patients had smaller metastatic lesions. The multivariable analysis shows that none of the clinical factors was significant, which indicated that the correlation between dose and lesion's volume should be recognized in the further research.

We analyzed RM and their related risk factors, and observed 1-year RM was 36.9%. Patients with oligoprogression were more likely to develop RM, and further treatment for those patients was still a question. Recently, a Japanese retrospective study assessed the safety and efficacy of repeat SBRT for 31 patients with local recurrence of stage I non-small-cell lung cancer or LM(which defined RM in our study). The first SBRT dose they used was 48–52 Gy/4F (*n* = 25), and the second doses were 48–52 Gy/4F or 60 Gy/8F, which were based on the tumor volume and the distance to organs at risk. Four patients showed no further recurrence for more than 5 years after the repeat SBRT, 4 patients suffered grade 2 radiation pneumonitis after the repeat SBRT, and no grade 3 pneumonitis was observed (25). In our study, 10 patients received repeat SBRT due to RM after the first course of SBRT, 2 patients exhibited local failure at the last follow up, and 1 of them showed grade 2 radiation pneumonitis, indicating the safety and efficacy of the second course of SBRT.

We observed strong correlation between the radiology evaluation around 5.3 months after SBRT and final LC rate. However, the suitable timing with regard to imaging response assessment was rarely reported on LM after SBRT. Sanuki et al. assessed the CT evaluations of tumor responses following SBRT for 42 hepatocellular carcinomas, whose SBRT dose was 35–40 Gy/5F. They demonstrated the median time to complete response (CR) defined by CT was 5.9 months, and CR increased from 10 (24%) to 28 (67%) to 30 (71%) tumors at 3, 6, and 12 months after SBRT (26). That interval was similar with our result. At the same time, in our study, 44 lesions with the status of PR/SD by CT examination didn't suffer local progression around 20 months, but most of those patients were all unsuitable for surgery or histologic puncture to confirm the pathology. Solanki et al. used PET-CT scan and CT based RECIST criteria for each treated lesion to evaluate response of 31 patients to SBRT. Of 22 patients with stable disease (SD) on CT scan, 13 achieved CR on PET, 8 achieved PR, and one still be judged SD. Of 21 metastases patients with PR by PET, 38% achieved CR, 52% remained PR, and 10% had progressive disease on follow-up PET (27). Thus, the metabolic factor was recommended for the persistent unincreased residual lesions after SBRT if necessary.

The main limitations of our study were the retrospective nature of the analysis. Selective bias was inevitable. For example, not all patients received gene detection or immunohistochemistry in order to make optimal systemic therapy, different prescription dose of SBRT was used, low BED accounted for a large proportion for fear of rib fracture or severe chest pain, and PET-CT scan or tissue biopsy were rarely performed for PR/SD patients. Further more, small population and short follow-up limited the confidence level of the consequence and we intend to launch a prospective study to validate our conclusion.

According to our results, SBRT can produce an effective LC and slightly toxicity for patients with OM or OP from CRC, and the GTV and BED can affect the LC although potential internal correlation between these two risk factors are needed in the future research. The occurrence rate of RM, tumor volume and adjacent normal tissue should be taken into consideration when making SBRT dose-fractionation schemes for patients with OM or OP from CRC, and 5.3 months after SBRT may represent the final response roughly although PET-CT scan or tissue biopsy are still needed for PR/SD patients.

## Data Availability Statement

The majority of the datasets generated for this study are included in the manuscript/supplementary files. The details of other datasets are available from the corresponding author on reasonable request.

## Ethics Statement

The studies involving human participants were reviewed and approved by Ethics Committee of Peking University Cancer Hospital and Institute. Written informed consent for participation was not required for this study in accordance with the national legislation and the institutional requirements.

## Author Contributions

WW and YL: conceptualization. SZ, HWu, and WW: methodology. SL: formal analysis. SL, DD, and JG: investigation. WW, YL, YC, and XZ: resources. SL, DD, and JG: data curation. SL: writing—original draft preparation. WW and YL: writing—review and editing. HWu and YC: supervision. CS, YZ, HWa, and XZ: project administration.

### Conflict of Interest

The authors declare that the research was conducted in the absence of any commercial or financial relationships that could be construed as a potential conflict of interest.
